# Financing Long-term Care: The Role of Culture and Social Norms: Comment on "Financing Long-term Care: Lessons From Japan"

**DOI:** 10.15171/ijhpm.2019.110

**Published:** 2019-11-11

**Authors:** Peter Alders, Frederik Schut

**Affiliations:** Erasmus School of Health Policy and Management, Erasmus University, Rotterdam, The Netherlands.

**Keywords:** Long-term Care Insurance, Public Insurance, Social Norms, Universal Coverage

## Abstract

Based on the experiences of Japan and Germany, Ikegami argues that middle-income countries should introduce public long-term care insurance (LTCi) at an early stage, before benefits have expanded as a result of ad hoc policy decisions to win popular support. The experience of the Netherlands, however, shows that an early introduction of public LTCi may not prevent, but instead even facilitate later extensions of public coverage. We argue that social norms and cultural values about caring for the elderly might be the main driver of expansions of LTCi coverage. Furthermore, we posit that this expansion may reinforce the social norms supporting it. Hence, politicians and policy-makers should be aware of this possible self-reinforcing effect.

## Introduction

In a recent editorial of this journal, Ikegami argues that middle-income countries which have achieved universal health coverage, should introduce public long-term care insurance (LTCi) at an early stage, before benefits have expanded as a result of ad hoc policy decisions to win popular support.^1^ His argument is based on a comparison of the introduction of public LTCi in Germany and Japan. In 1995 Germany implemented a public LTCi scheme with relatively limited services and an option for cash benefits. According to Ikegami, Germany was able to hold off major expansions of public LTCi because at the time of introduction public coverage of long-term care (LTC) services was largely absent. By contrast, in 2000 Japan only introduced public LTCi after access to LTC services was created by ad hoc expansions of public health insurance coverage (eg, long-stays in hospitals) and the social welfare schemes. Hence, Japan started with a more generous LTCi scheme than Germany, which made it much more difficult to contain costs. The ability to contain LTC costs may be especially relevant given that in many societies LTC expenditure is likely to grow due to a rapidly ageing population.

Based on the experience of the Netherlands, however, we question whether an early introduction of public LTC insurance is really key to restricting the expansion of publicly financed LTC services. In 1968, the Netherlands was by far the first country to implement a public LTC insurance scheme. Although initially only a limited number of LTC services were covered, the scope of benefits was steadily expanded over time. Eventually, this resulted in a much more generous scheme than the Japanese, with a level of public spending on LTC (as percentage of gross domestic products) that is among the highest in the world.^2^ Clearly, the early introduction of public LTCi did not prevent this. Hence, the key question is why the Germans were successful in preventing an expansion of public LTC insurance, while the Dutch were not? And what lessons can be learned by middle-income countries contemplating about establishing public LTCi?

Our proposition is that the social norms about caring for elderly were of crucial importance for the moment of introduction of public LTCi and its development. We argue that the introduction of public LTCi and the successive expansions reinforced the culturally based social norms that supported its early introduction.

Before discussing our proposition in more detail, we first provide a brief description of the evolution of the Dutch public LTC insurance scheme, in line with the description of the German and Japanese schemes by Ikegami.^1^

## Long-term Care Insurance in The Netherlands

In 1968, the Netherlands introduced a universal mandatory social health insurance scheme by adoption of the Exceptional Medical Expenses Act (abbreviated as AWBZ). There are several reasons why in the Netherlands the choice was made for a universal public health insurance scheme for LTC.^3^ Prior to 1968 the financing of LTC facilities was highly fragmented and increasingly insufficient to provide access to adequate care for lower-income groups. The strong economic growth during the 1960s substantially increased the general welfare of society. Since the financial risk of LTC was considered to be largely uninsurable on a private market, there was broad political support to expand public financing to cover this risk. Moreover, this was in line with the general social support for a rapidly expanding welfare state that would take care of the population from cradle to grave. Initially, the AWBZ covered primarily nursing home care, institutionalized care for the mentally handicapped, and hospital admissions lasting more than a year. In due course, however, coverage was expanded by including home healthcare, eg, for rehabilitation at home after hospital admission and care for elderly people with impairments (in 1980), ambulatory mental healthcare (in 1982), family care, eg, home help in case of frailty, psychosocial problems or after childbirth (1989) and residential care for the elderly (1997). Contributions and co-payments were earmarked and income-related and collected in a national insurance fund, from which providers were paid. In 1995 cash benefits were introduced, which eligible people can substitute for service benefits.

## The Role of Culture and Social Norms

The implementation of public LTCi cannot be seen apart from social norms and cultural values. For instance, a negative relationship is found between family ties and expected coverage of LTCi.^4^ Therefore, investigating social norms might add to the explanation why the Netherlands were earlier than other countries to introduce LTC insurance. According to Hofstede’s comparative model of national culture, in which 6 different cultural dimensions are distinguished, the Netherlands is characterized by a highly individualistic and feminine society, whereas the opposite holds for Japan, and Germany being in between both countries (see Figure).^5^ In individualist societies people are supposed to look after themselves and their direct family only. By contrast, in collectivist societies people belong to ‘in groups’ that take care of them in exchange for loyalty. Feminine countries are inclusive and people value equality, solidarity and quality in their working lives. In feminine societies caring for others and solidarity are dominant values, but the family structure is more flexible than the more traditional family structure in masculine countries. Hence, although the demand for care for frail people is felt in society, it could be less automatic that provision of this care is carried out within the family. The combination of individualism and social solidarity in the Netherlands may well explain the strong social support for extensive welfare state arrangements and the early adoption and subsequent expansion of a universal public LTC insurance scheme. Interestingly, a similar culture of individualism and femininity is observed in Sweden, which is characterized by equally comprehensive publicly financed LTC arrangements.

**Figure:**
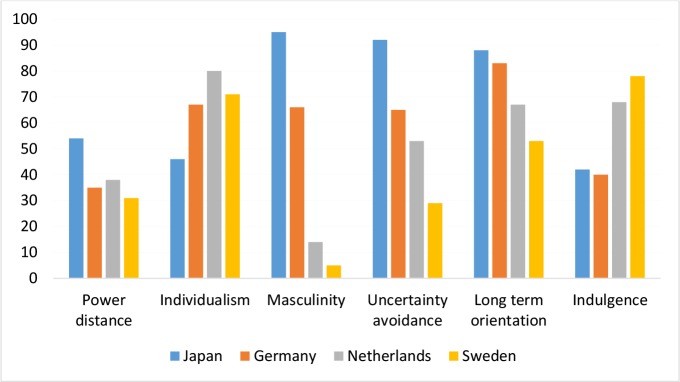
Index of 6 Cultural Dimensions in Japan, Germany, the Netherlands, and Sweden. Source: https://www.hofstede-insights.com/product/compare-countries/.

A comparative study on the provision of elderly care in Europe found that in the Scandinavian countries and the Netherlands the state is regarded as being responsible for providing care, while there are only weak legal obligations for relatives to provide informal care.^6^ In the countries with family-based care systems, like Japan, Germany, Austria and most of Mediterranean countries, the responsibility for the care of an older person with needs is primarily borne by their relatives.^6,7^ Compared to the Netherlands, in Germany a much larger share of the respondents (40.7% vs. 5.3%) of the Eurobarometer questionnaire of 2007 stated that parents should live with their children “when an elderly father or mother who lives alone and can no longer manage to live without regular help because of her or his physical or mental health condition.”^8^ By contrast, in the Netherlands more people responded that they should move to a nursing home (46.3% vs. 22.4%). Nevertheless, the preferences for institutional care in the Netherlands are slowly changing, as it became easier and more attractive to live longer independently in the community. Consequently, the frailty of the population in nursing homes increased, which may have contributed to a deterioration of the attractiveness of institutional care.^9^

Once present, the Dutch public LTCi scheme may have reinforced the culture of individualism and related social norms about caring for the elderly population. The increasing access and availability of home and nursing home care made children less prepared taking responsibility for caring for their parents when getting old and in need of care. Hence, letting a parent go to an elderly home or being assisted by professional home help and community nurses became the socially acceptable default option.^8^ The broad availability of formal LTC services may also have influenced the expectations of older adults about receiving those services when they get more dependent. The same might be true for housing policies in the past. In the Netherlands, since World War II older adults have been nudged to “elderly homes” to combat the postwar housing shortage.^8^ Furthermore, over time the entire LTC provision in the Netherlands became oriented towards older people becoming depended on publicly provided professional and institutional LTC services.

This has created a strong vested interest in maintaining and supporting the prevailing social norm. By contrast, the different family-based (masculine) social norms in Germany might explain why a strong expansion of public LTCi did not happen there, once the rather parsimonious public LTCi scheme was introduced in 1995. The reason why the public LTCi scheme in Japan has been expanded despite the prevailing strong masculine social norms, may be explained by a combination of strong social obligations for the family to provide elderly care and a rapidly ageing population.^7^ As pointed out by Nakabayashi^7^ the expansion of the public LTCi scheme in Japan may have produced a net social welfare gain, as the benefits of alleviating families from the high burden of LTC provision may have outweighed the additional public expenditure on LTC.

## Policy Implications

The Dutch experience show that an early adoption of public LTCi is no guarantee to prevent or slowdown an expansion of benefits covered. When this expansion is in line with the prevailing social norms and cultural values, an early adoption of public LTCi may even facilitate such an expansion because the introduction of publicly financed LTC may reinforce the prevailing social norms.

In view of an ageing population, however, the last decade Dutch policy-makers became increasingly worried about the financial sustainability of the generous public LTCi scheme. The call for cost containment became stronger, especially after the severe economic recession of 2008, resulting in an intensifying societal debate on shifting the public responsibility for the provision of LTC partly to the citizens and their families and social network. Eventually, after 10 to 20 years discussions and smaller policy measures to contain public LTC expenditure, in 2015 this resulted in a major LTC reform by which the coverage of the public LTCi scheme was restricted to institutionalized care and intensive (24 hour) home healthcare.^10,11^ Coverage of less intensive home care was transferred to the social health insurance scheme, while municipalities became responsible for providing social LTC services. Especially for social LTC services people’s own responsibility has been reinforced, since municipalities are only legally obliged to provide care if people’s family and social network cannot adequately support them. Although there is some weak evidence that the reform may have had some impact on the social norms concerning the provision of LTC, these norms tend to be quite robust and difficult to change.^12^ Moreover, despite the major change in the way LTC is financed, the extent of public provision of LTC has hardly been reduced.

## Conclusion

Ikegami posits that an early introduction of public LTCi can prevent ad-hoc extensions. The Dutch experience shows, however, that an early introduction may not prevent but instead even facilitate such extensions. Prevailing social norms may well be the main driver of extensions of public coverage, and are likely to be reinforced when the burden for informal caregivers is high. Furthermore, we posit that the broad availability of publicly financed LTC is likely to have a perpetuating effect on the social norms supporting the extensions. Hence, particularly in middle income countries, politicians and policy-makers should be aware of this possible self-reinforcing effect, and have to weigh this against the social welfare gains from alleviating the burden for informal caregivers.

## Acknowledgements

We thank an anonymous reviewer for valuable comments on a previous draft.

## Ethical issues

Not applicable.

## Competing interests

Authors declare that they have no competing interests.

## Authors’ contributions

Both authors contributed to the concept and writing of the article.
